# Prospective Comparative Study of Dysphagia after Subaxial Cervical Spine Surgery: Cervical Spondylotic Myelopathy and Posterior Longitudinal Ligament Ossification

**DOI:** 10.3390/jcm12051774

**Published:** 2023-02-23

**Authors:** Kyohei Sakaki, Kenichiro Sakai, Yoshiyasu Arai, Ichiro Torigoe, Masaki Tomori, Takashi Hirai, Hiroaki Onuma, Yutaka Kobayashi, Atsushi Okawa, Toshitaka Yoshii

**Affiliations:** 1 Department of Orthopedic Surgery, Saiseikai Kawaguchi General Hospital, 5-11-5 Nishikawaguchi, Kawaguchishi 332-8558, Japan; 2 Department of Orthopaedics, Graduate School, Tokyo Medical and Dental University, Tokyo 108-0075, Japan

**Keywords:** ossification of the posterior longitudinal ligament, cervical spondylotic myelopathy, surgical complication, postoperative dysphagia

## Abstract

We prospectively investigated the postoperative dysphagia in cervical posterior longitudinal ligament ossification (C-OPLL) and cervical spondylotic myelopathy (CSM) to identify the risk factors of each disease and the incidence. A series of 55 cases with C-OPLL: 13 anterior decompression with fusion (ADF), 16 posterior decompression with fusion (PDF), and 26 laminoplasty (LAMP), and a series of 123 cases with CSM: 61 ADF, 5 PDF, and 57 LAMP, were included. Vertebral level, number of segments, approach, and with or without fusion, and pre and postoperative values of Bazaz dysphagia score, C2-7 lordotic angle (∠C2-7), cervical range of motion, O-C2 lordotic angle, cervical Japanese Orthopedic Association score, and visual analog scale for neck pain were investigated. New dysphagia was defined as an increase in the Bazaz dysphagia score by one grade or more than one year after surgery. New dysphagia occurred in 12 cases with C-OPLL; 6 with ADF (46.2%), 4 with PDF (25%), 2 with LAMP (7.7%), and in 19 cases with CSM; 15 with ADF (24.6%), 1 with PDF (20%), and 3 with LAMP (1.8%). There was no significant difference in the incidence between the two diseases. Multivariate analysis demonstrated that increased ∠C2-7 was a risk factor for both diseases.

## 1. Introduction

Postoperative dysphagia is one of the most common complications of anterior cervical spine surgery and is known to occur at a high rate [1,2,3,4,5,6]. Although it has been reported in recent years that it also occurs in posterior cervical spine surgery, the incidence has varied widely [7,8,9], and there is not enough information on the exact incidence or the risk factors.

Ossification of the posterior longitudinal ligament of the cervical spine (C-OPLL) is one of the degenerative spinal diseases causing neuropathy in middle-aged and older patients [10,11,12], and the prevalence of C-OPLL in East Asian countries ranges from 1.9% to 4.3% [13,14]. Considerable risk of complications is typically linked with surgical treatment of C-OPLL with neuropathy [15,16,17,18]. C-OPLL is related to a higher complication rate of ossification of the anterior longitudinal ligament than cervical spondylotic myelopathy (CSM), and is thought to be more often involved in dysphagia due to decreased mobility caused by ossification [19,20], but there are few prospective research studies on the incidence and risk factors of postoperative dysphagia focusing on C-OPLL. A prospective comparative study of the incidence and risk factors for postoperative dysphagia in C-OPLL and CSM was performed to characterize postoperative dysphagia in C-OPLL.

## 2. Materials and Methods

Prospective cases included the 202 consecutive C-OPLL or CSM patients who underwent subaxial cervical spine surgery at our department from April 2015. Patients with upper cervical spine surgery, combined anterior and posterior cervical surgery, corrective surgery for cervical deformity, surgery for trauma, infection, or tumor, and a history of previous cervical spine surgery were excluded in advance. Incomplete data for 13 patients, 2 patients who received additional cervical spine surgery, and 9 patients who were lost to follow-up resulted in the exclusion of 24 individuals from the study. Finally, 55 C-OPLL and 123 CSM patients were included.

For patients with CSM, laminoplasty (LAMP) was chosen if the following three criteria were met, and fusion was selected if any of these criteria were not met: (1) no kyphosis in cervical alignment; (2) center of gravity of the head to C7 sagittal vertical axis (CGH-C7SVA) < 40 mm; and (3) no intervertebral instability. For patients with C-OPLL, LAMP was indicated when the following five criteria were met, and fusion was indicated when any one of the these criteria were not met: (1) no kyphosis in cervical alignment; (2) CGH-C7SVA < 40 mm; (3) no intervertebral instability; (4) K-line(+); and (5) canal occupancy ratio < 50%. Patients who required fusion underwent anterior cervical discectomy with fusion for CSM and anterior corpectomy and fusion for C-OPLL, but posterior decompression and fusion (PDF) were selected for patients with respiratory disorder or those who required multilevel surgeries despite their poor general condition. In all cases with anterior decompression and fusion (ADF), a surgical approach was made from the left side and cervical reconstruction was conducted with cage or autologous iliac/fibula bone graft and plate-and-screw system fixation. Except for flattening the vertebral body in preparation for plate placement, no aggressive osteophyte removal was done. PDF was performed using laminectomy and instrumented fixation with local bone graft, and cervical fixation was performed with the screw-and-rod system at the decompressed segments. LAMP was done using the double-door system without spacers by Miyazaki and Kirita [21].

The Bazaz dysphagia score (Table 1) was used to test all 178 patients for dysphagia before, 6 months after, and 1 year following surgery. The Bazaz dysphagia score after surgery worsened by at least one grade to be considered new dysphagia. Levels of surgery, number of operative segments, surgical approach (anterior or posterior), with or without fixation, and pre and postoperative values and the amount of change in C2-7 lordotic angle (∠C2-7), C2-7 range of motion (ROM), O-C2 lordotic angle (∠O-C2) were examined. Clinical results were assessed using the Japanese Orthopedic Association scoring system for cervical myelopathy (C-JOA score) and neck pain visual analog scale (VAS). Using the Hirabayashi approach, the recovery rate of the C-JOA score was computed [22]. Examined and compared between C-OPLL and CSM were the occurrences of new dysphagia at 6 months and 1 year postoperatively for each procedure, PDF, LAMP, and ADF. We divided all patients into the New Dysphagia group and the Non-new Dysphagia group with or without new dysphagia at one-year postoperatively and compared each preoperative and operative factor between the two groups by univariate analysis to examine the risk factors of persistent dysphagia in CSM and C-OPLL, respectively. To analyze more influent risk factors, multivariate analyses were conducted with the risk factors listed in the univariate analysis as explanatory variables and new dysphagia at one-year postoperatively as the objective variable. The threshold for the occurrence of persistent new dysphagia was calculated using receiver operating characteristic (ROC) analysis.

Student’s *t*-test, Fisher’s exact test, χ-square test, Mann–Whitney U test, ROC analysis, and logistic regression analysis were employed for statistical analysis. EZR was utilized as the statistical software [23]. The study was approved by the Ethics Committee of our institution.

## 3. Results

Table 2 displays demographic information on patients before surgery. There was no difference in age, gender, C-JOA score, and neck pain VAS between C-OPLL and CSM patients, but C-OPLL patients had a significantly wider operative range, smaller ∠C2-7 and ROM, and larger ∠O-C2 compared to CSM patients. For the 55 cases of C-OPLL, ADF was conducted in 13 cases, PDF in 16 cases, and LAMP in 26 cases; for the 123 cases of CSM, ADF was carried out in 61 cases, PDF in 5 cases, and LAMP in 57 cases (Table 3).

Table 4 displays the degree of dysphagia as determined by the Bazaz dysphagia score and the total number of symptomatic patients at each observational time point. Eleven patients with C-OPLL and twenty-one patients with CSM already had dysphagia before surgery, and there was no significant difference in the prevalence of both diseases (*p* = 0.796). At 6 months postoperatively, 14 cases (25.5%) of C-OPLL and 24 cases (19.5%) of CSM had created new dysphagia, of which 12 cases (21.8%) of C-OPLL and 19 cases (15.4%) of CSM continued to have dysphagia until one-year postoperatively. In posterior approach methods, new dysphagia of four cases (25%) with PDF and two cases (7.7%) with LAMP in C-OPLL and one case (20%) with PDF, and three cases (1.8%) with LAMP in CSM remained until 1 year postoperation. At each observational time point, there was no significant difference in the incidence of new dysphagia for any procedure or both illnesses (Table 5). Some of the new dysphagia found at 6 months in patients with ADF improved at 1 year postoperatively, but all new dysphagia seen at 6 months in PDF and LAMP patients persisted to 1 year after surgery.

Comparing preoperative and operative factors between the New Dysphagia and the Non-new Dysphagia groups in C-OPLL, the New Dysphagia group had significantly higher rates of anterior method and fixation surgery. Postoperative ∠C2-7 and change in ∠C2-7 were significantly higher in the New Dysphagia group, and change in ∠O-C2 was significantly lower. The New Dysphagia group in the CSM was much younger, had a more anterior approach and fixation surgeries, and had a bigger shift in the ∠C2-7 (Table 6). Increased∠C2-7 was the only risk factor for new dysphagia for both C-OPLL and CSM, according to multivariate analyses (Table 7).

**Figure 1:**
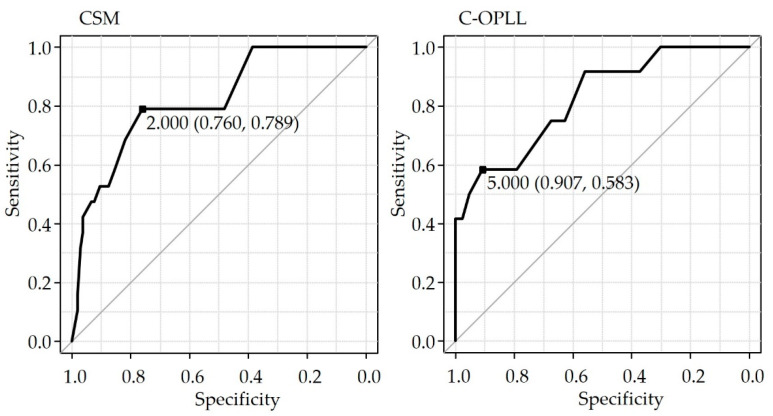
ROC curve. C-OPLL, Ossification of the posterior longitudinal ligament of the cervical spine; CSM, cervical spondylotic myelopathy.

ROC analysis with new dysphagia as the objective variable and increase in ∠C2-7 as the explanatory variable demonstrated that the threshold for CSM was 2° with a sensitivity of 0.789 and specificity of 0.760 (area under the curve was 0.817, 95% confidence interval 0.713–0.921). The threshold for C-OPLL was 5°, with a sensitivity of 0.583 and specificity of 0.907 (area under the curve: 0.827, 95% confidence interval: 0.686–0.961) (Figure 1).

## 4. Discussion

It has been reported that the incidence of postoperative dysphagia is extremely low in posterior cervical spine surgery compared to anterior surgery [24,25]. However, there are some reports that postoperative dysphagia has been noted in 9% to 21% of patients even in posterior cervical surgery [7,8,9], and it is now understood that this consequence is not specific to anterior cervical surgery. Postoperative dysphagia after anterior cervical surgery is known to be one of the most frequent issues, reported to happen at a very high rate of 52.9–63.6% [26,27], while others report that it occurs in only 1.9% [28]. One of the causes of these kinds of the disparity in incidence in anterior surgery and the underestimation of postoperative dysphagia in posterior surgery is thought to be the inconsistencies in the definition of dysphagia as a complication [29].

The incidence of dysphagia differs depending on whether the observation is early or late, postoperatively [7,8]. Swelling of the anterior soft tissue of the vertebral body following ACDF can last for up to 6 months [30], and perioperative administration of dexamethasone significantly improves the immediate postoperative edema of the anterior soft tissue of the vertebral body but does not affect long-term dysphagia [31]. Based on the results, it could be hypothesized that the mechanism of early and late postoperative dysphagia is different and that the postoperative dysphagia in the early period especially in anterior surgery includes the effects of direct anterior soft-tissue involvement. In the current study, new dysphagia noticed 6 months after posterior surgery persisted until one year after surgery, but 7 of 28 cases (25%) with new dysphagia were observed at 6 months after ADF improved at one year postoperative. This result indicates that more than 6 months of follow-up is important to examine persistent dysphagia after anterior surgery. Previously observed risks for dysphagia after anterior cervical surgery include smoking [32,33,34], female [32,33], preoperative dysphagia [32,35], prior cervical spine surgery [34], multilevel surgeries [27,33,34,36,37], duration of surgery [33,38], traction esophagus [26,35], bone morphogenetic protein-2 use [39], and recurrent laryngeal nerve damage [40], some of which may include risk of transient dysphagia due to direct invasion to anterior tissue of the cervical spine. In this prospective research based on a consistent definition of dysphagia, 6 cases (46.2%) with C-OPLL and 15 cases (24.6%) with CSM in anterior surgery, and 6 cases (14.3%) with C-OPLL and 4 cases (6.5%) with CSM in posterior surgery had postoperative dysphagia at one year after surgery. This implies that even with posterior surgery, postoperative dysphagia occurs frequently.

The presence of anterior cervical osteophytes, which are frequently combined in patients with C-OPLL [41], causes progressive dysphagia, discomfort of pharynx, and odynophagia [42,43], and their impact is supposed to depend on the size and localization of the osteophytes [44,45,46,47]. Additionally, severe dysphagia brought on by osteophytes is thought to be successfully treated by surgically removing hyperplastic osteophytes [48,49,50]. These would indicate a higher rate of postoperative dysphagia in C-OPLL, but in fact, there are few reports on the effect of anterior osteophytes on postoperative dysphagia, and Chin et al. found that preoperative osteophyte height does not affect postoperative dysphagia after ACDF [51]. It is also recognized that normal swallowing is accompanied by the mobility of the cervical spine [52,53], and Morishita et al. supposed that decreased mobility of the cervical spine might be one of the reasons for postoperative dysphagia after laminoplasty for C-OPLL [20], but no unified view has been collected so far regarding the influence of cervical mobility. In this study, the incidence of new dysphagia in C-OPLL was not significantly different from that in CSM, either in total or by procedure, and it did not indicate that postoperative dysphagia is more common in C-OPLL than in CSM.

The most influential risk factor for postoperative dysphagia was an increase of ∠C2-7 in both CSM and C-OPLL. The male-female ratio did not affect the occurrence of postoperative dysphagia in this comprehensive prospective study, although it seems to be independent of the surgical procedure and follow-up period among the known risk factors. It was previously found that an increase in lordotic angle of >5° is a risk factor for dysphagia in anterior surgery [54], and recently it has been indicated that it is also a risk factor in posterior surgery [9,55]. It is well known that a decrease in the O-C2 lordotic angle in head and neck junction surgery could cause postoperative dysphagia, and it has been reported that the primary site of dysphagia is an anterior protrusion of the subaxial cervical spine [56]. The pharyngeal and esophageal cavities may constrict with increasing postoperative lordotic angle in subaxial cervical spine surgery, which may lead to dysphagia. Especially, since the cervical lordosis angle is permanent after surgery in-fixation surgery, surgical planning to avoid making excessive lordosis and check of intraoperative lordotic angle may avoid unexpected postoperative dysphagia.

This study has the following limitations. First, these surgeries were conducted by multiple surgeons, although the choice of surgical method is partially dependent on the surgeon. Second, the number of cases involved is small. Third, this study was intended to explore the risk factors of persistent dysphagia, but a one-year study period may not be sufficient. Additionally, few patients with severe dysphagia were included in this study, and further clinical studies might be required to examine the risk factors for severe dysphagia. However, this is the first comprehensive prospective study for postoperative dysphagia in C-OPLL. These findings may help surgeons prevent dysphagia after C-OPLL surgery.

## Figures and Tables

**Table 1:** Bazaz dysphagia score.

Severity	Symptoms	
	Liquid	Solid
None	None	None
Mild	None	Rare
Moderate	None or rare	Occasionally (only with specific food)
Severe	None or rare	Frequent (majority of solids)

**Table 2:** Patient demographic data.

	C-OPLL	CSM	*p*
Number of cases	55	123	
Age at surgery, years	63.4 ± 13.4	67.2 ± 12.3	0.063
Gender (male:female)	42:13	80:43	0.163
Upper vertebra of operative segments	2.8 ± 0.7	3.7 ± 1.0	0.000 *
Lower vertebra of operative segments	6.7 ± 0.8	6.4 ± 1.0	0.038 *
No. of operative segments	3.9 ± 1.2	2.7 ± 1.2	0.000 *
C-JOA score (pts)	11.2 ± 2.3	11.5 ± 2.7	0.391
∠C2-7 (°)	11.5 ± 12.6	15.4 ± 12.1	0.05 *
∠O-C2 (°)	20.5 ± 7.5	17.2 ± 8.4	0.013 *
ROM (°)	32.6 ± 12.7	40.7 ± 13.5	0.000 *
Neck pain (VSA mm)	46.2 ± 30.5	41.3 ± 32.3	0.345

C-OPLL, Ossification of the posterior longitudinal ligament of the cervical spine; CSM, cervical spondylotic myelopathy; C-JOA, Japanese Orthopedic Association for cervical myelopathy; ∠C2-7, C2-7 lordotic angle; ∠O-C2, O-C2 lordotic angle; ROM, C2-7 range of motion; VAS, visual analog scale. * *p* < 0.05.

**Table 3:** Operative procedure.

	ADF	PDF	LAMP
C-OPLL (cases)	13	16	26
CSM (cases)	61	5	57

ADF, anterior decompression and fusion; PDF, posterior decompression and fusion; LAMP, laminoplasty; C-OPLL, Ossification of the posterior longitudinal ligament of the cervical spine; CSM, cervical spondylotic myelopathy.

**Table 4:** Bazaz dysphasia score at each observation point serves to determine the status of dysphagia.

	C-OPLL			CSM		
Bazaz dysphasia score	Pre	6 M	1 Y	Pre	6 M	1 Y
None (cases)	44	36	37	102	92	97
Mild (cases)	10	16	15	19	23	20
Moderate (cases)	1	2	2	2	7	6
Severe (cases)	0	1	1	0	1	0

C-OPLL, Ossification of the posterior longitudinal ligament of the cervical spine; CSM, cervical spondylotic myelopathy; M, monhs; Y, year.

**Table 5:** New dysphagia.

	6 M				1 Y			
	ADF	PDF	LAMP	Total	ADF	PDF	LAMP	Total
C-OPLL	8 (61.5%)	4 (25%)	2 (7.7%)	14 (25.5%)	6 (46.2%)	4 (25%)	2 (7.7%)	12 (21.8%)
CSM	20 (32.8%)	1 (20%)	3 (1.8%)	24 (19.5%)	15 (24.6%)	1 (20%)	3 (1.8%)	19 (15.4%)
*p* value	0.231	1	0.646	0.489	0.173	1	0.646	0.411

M, monhs; Y, year; ADF, anterior decompression and fusion; PDF, posterior decompression and fusion; LAMP, laminoplasty; C-OPLL, Ossification of the posterior longitudinal ligament of the cervical spine; CSM, cervical spondylotic myelopathy.

**Table 6:** Preoperative and surgical factors in the new dysphagia and non-new dysphagia groups —comparative analysis.

	C-OPLL			CSM		
	Non-New Dysphagia	New Dysphagia	*p*	Non-New Dysphagia	New Dysphagia	*p*
Number of cases	43	12		104	19	
Preoperative factors						
Predysphagia score (cases)	None: 33 Mild: 9 Moderate: 1	None:11 Mild:1	0.259	None: 85 Mild: 17 Moderate: 2	None: 17 Mild: 2	0.403
Age at surgery (yrs)	63.4 ± 13.8	63.3 ± 12.3	0.965	68.2 ± 12.2	62.0 ± 12.0	0.043 *
Gender (male:female)	33:10	9:3	1	65:39	15:4	0.199
C-JOA score (pts)	11.3 ± 2.0	10.6 ± 3.1	0.325	11.4 ± 2.7	12.2 ± 3.1	0.209
∠C2-7 (°)	12.1 ± 12.3	9.4 ± 13.0	0.516	16.2 ± 11.8	11.2 ± 13.1	0.097
∠OC2 (°)	20.7 ± 7.4	19.8 ± 7.9	0.734	17.3 ± 8.5	16.4 ± 7.6	0.673
ROM (°)	32.8 ± 13.0	31.8 ± 12.2	0.816	40.4 ± 13. 9	42.6 ± 10.7	0.526
Neck pain (VSA mm)	45.0 ± 30.0	50.4 ± 34.8	0.59	43.0 ± 33.2	32.2 ± 26.2	0.184
Operative factors						
Approach (anterior cases: posterior cases)	7:36	6:6	0.024 *	46:58	15:4	0.006 *
Fusion surgery (fusion cases: nonfusion cases)	19:24	10:2	0.023 *	50:54	16:3	0.005 *
No. of operative segments	3.9 ± 1.2	3.8 ± 1.4	0.759	2.7 ± 1.3	2.6 ± 1.1	0.739
Upper vertebra	2.9 ± 0.7	2.7 ± 0.9	0.302	3.7 ± 1.0	3.8 ± 1.0	0.678
Lower vertebra	6. 8 ± 0.8	6.4 ± 0.8	0.207	6.4 ± 1.0	6.4 ± 0.9	0.813
Postoperative factors						
Post C-JOA score (pts)	13.7 ± 2.0	14.2 ± 1.7	0.609	13.8 ± 2.2	14.7 ± 2.2	0.0614
Recovery rate of C-JOA score (%)	43.2 ± 28.9	54.0 ± 24.8	0.275	40.9 ± 37.7	57.3 ± 31.5	0.0811
Post ∠C2-7 (°)	7.1 ± 10.0	15.2 ± 10.2	0.017 *	15.1 ± 12.0	18.2 ± 9.7	0.294
⊿∠C2-7 (°)	−5.00 ± 7.0	5.8 ± 9.3	0.004 *	−1.09 ± 5.9	7.0 ± 6.4	0.000 *
Post ∠OC2 (°)	23.9 ± 9.3	18.0 ± 10.9	0.066	16.8 ± 8.4	14.7 ± 8.0	0.323
⊿∠OC2 (°)	3.2 ± 5.5	−1.8 ± 9.9	0.024 *	−0.5 ± 5.0	−1.7 ± 4.3	0.333
Post ROM (°)	18.7 ± 13.9	16.3 ± 14.0	0.591	31.1 ± 12.0	25.8 ± 14.4	0.093
⊿ROM (°)	−14.1 ± 15.4	−12.9 ± 11.8	0.804	−9.3 ± 14.9	−18.2 ± 13.1	0.019
Post neck pain (VAS mm)	43.8 ± 30.1	49.5 ± 29.3	0.578	35.0 ± 29.9	36.8 ± 33.5	0.827
⊿Neck pain (VAS mm)	−1.2 ± 36.4	−4.5 ± 29.3	0.778	−7.9 ± 36.3	0.8 ± 22.1	0.344

⊿ indicates postoperative data—preoperative data; C-OPLL, Ossification of the posterior longitudinal ligament of the cervical spine; CSM, cervical spondylotic myelopathy; ∠C2-7, C2-7 lordotic angle; C-JOA, Japanese Orthopedic Association for cervical myelopathy; ∠OC2, O-C2 lordotic angle; ROM, C2-7 range of motion; VAS, visual analog scale. * *p* < 0.05.

**Table 7:** Risk factors of new dysphagia at 1-year after surgery.

CSM			
	*p*	OR	95% CI
Age	0.739	-	-
Approach	0.679	-	-
Fusion surgery	0.258	-	-
⊿C2-7	0.000 *	1.20	1.09–1.32
**C-OPLL**			
	***p***	**OR**	**95% CI**
Approach	0.647	-	-
Fusion surgery	0.193	-	-
⊿C2-7	0.017 *	1.20	1.03–1.40
Post C2-7	0.102	-	-
⊿∠OC2	0.999	-	-

⊿ indicates postoperative data—preoperative data; C-OPLL, Ossification of the posterior longitudinal ligament of the cervical spine; CSM, cervical spondylotic myelopathy; CI, confidence interval; CL, C2-7 lordotic angle; ∠OC2, O-C2 lordotic angle; OR, odd ratio. * *p* < 0.05.

## Data Availability

Detailed data are available from the first author upon request.
